# National point prevalence survey on healthcare-associated infections in acute care hospitals, Switzerland, 2017

**DOI:** 10.2807/1560-7917.ES.2019.24.32.1800603

**Published:** 2019-08-08

**Authors:** Walter Zingg, Aliki Metsini, Carlo Balmelli, Dionysios Neofytos, Michael Behnke, Céline Gardiol, Andreas Widmer, Didier Pittet, on behalf of the Swissnoso Network

**Affiliations:** 1 Infection Control Programme and WHO Collaborating Centre on Patient Safety, University of Geneva Hospitals and Faculty of Medicine, Geneva, Switzerland; 2 These authors contributed equally; 3 Infection Control Programme, Cantonal Hospital Authority, Ticino, Switzerland; 4 Institute of Hygiene and Environmental Medicine, Charité University Medicine Berlin, Berlin, Germany; 5 Swiss Federal Office of Public Health, Bern, Switzerland; 6 Division of Infectious Diseases and Hospital Epidemiology, University Hospital Basel, Switzerland; 7 Members of the Swissnoso Network are acknowledged at the end of this article

**Keywords:** Point prevalence Survey, Switzerland, healthcare-associated infection, acute care, Swissnoso, ECDC

## Abstract

**Background:**

As a part of the national strategy on the prevention of healthcare-associated infections (HAI), a point prevalence survey (PPS) was conducted in acute care hospitals in Switzerland.

**Aim:**

Our objective was to assess the burden of HAI in Swiss acute care hospitals.

**Methods:**

All acute care hospitals were invited to participate in this cross-sectional survey during the second quarter of 2017. The protocol by the European Centre for Disease Prevention and Control was applied. Patients of all ages, hospitalised on the day of survey were included, except when admitted to outpatient clinics, emergency and psychiatry.

**Results:**

Ninety-six acute care hospitals (79% of all hospitals ≥ 100 beds) provided data on 12,931 patients. Pooled and randomised HAI prevalences were 5.9% (95% confidence interval (CI): 5.5–6.3) and 5.4% (95% CI: 4.8–6.0), respectively. The HAI incidence was estimated at 4.5 (95% CI: 4.0–5.0). The most common type of HAI was surgical site infection (29.0%), followed by lower respiratory tract (18.2%), urinary tract (14.9%) and bloodstream (12.8%) infections. The highest prevalence was identified in intensive care (20.6%), in large hospitals > 650 beds (7.8%), among elderly patients (7.4%), male patients (7.2%) and patients with an ultimately (9.3%) or rapidly (10.6%) fatal McCabe score.

**Discussion:**

This is the first national PPS of Switzerland allowing direct comparison with other European countries. The HAI prevalence was at European Union average (5.9% in 2016 and 2017), but higher than in some countries neighbouring Switzerland. Based on the limited information from previous surveys, HAI appear not to decrease.

## Introduction

The occurrence of healthcare-associated infections (HAI) is a preventable patient safety concern [1,2]. An estimated 2.7 million new HAI cases occur every year in the European Union (EU) and the European Economic Area (EEA) together, contributing to a cumulative burden of 501 disability-adjusted life years per 100,000 general population [3]. Surveillance of HAI incidence with timely feedback has become standard in HAI prevention [4]. However, because of the high work effort, prospective HAI surveillance is mostly oriented towards high prevalence areas such as intensive care units or is confined to specific infection types such as surgical site infection (SSI) [5,6] or bloodstream infection (BSI) [7].

As an alternative, point-prevalence surveys (PPS) have been performed for many years to estimate the burden of HAI [8,9]. The “Study on the Efficacy of Nosocomial Infection Control” (SENIC) by the United States (US) Centers for Disease Control and Prevention (CDC), applied repeated PPS to assess the effectiveness of infection prevention and control (IPC) programmes in acute care hospitals in the US [10]. Although challenging by its cross-sectional design [11], PPS offer a valid perspective on the hospital-wide burden of HAI. In 2011 and 2012, the European Centre for Disease Prevention and Control (ECDC) conducted its first PPS in the EU Member States, Iceland, Norway and Croatia [12] and 5 years later, it conducted its second PPS in the EU and EEA countries as well as in some EU candidate countries [13,14]. Switzerland, however, did not participate in these surveys.

Switzerland had performed national period prevalence surveys in the past, for the last time in 2003 and 2004 [15,16]. In January 2013, the Swiss Federal Council passed its ‘Health 2020’ agenda [17], which sets priorities in healthcare management in Switzerland. The prevention of HAI was identified as a first order measure and therefore, the Federal Office of Public Health (FOPH), together with stakeholders from various areas of health delivery in Switzerland, defined a national strategy on HAI prevention [18]. The strategy, called ‘Strategie NOSO’, aims at reducing HAI and containing the emergence and spread of antimicrobial resistance in the various healthcare settings in Switzerland. FOPH mandated Swissnoso to perform a national PPS (CH-PPS) on HAI in 2017 to estimate the burden of HAI in Swiss acute care hospitals. Swissnoso is a publicly funded association of experts in the field of IPC and infectious diseases in Switzerland (www.swissnoso.ch). The goal of this survey was to estimate the burden of HAI using the ECDC protocol and to compare the results with other European countries.

## Methods

### Setting and study population

In 2016, a pilot survey was performed in three large acute care hospitals in Switzerland with the objective to test the ECDC PPS protocol [19]. The CH-PPS protocol (www.swissnoso.ch/prevalence) was translated into the three major national languages (French, German and Italian) from the original English ECDC protocol version 5.3 [13]. In December 2016, all 187 acute care hospitals in Switzerland were invited to participate in the Swissnoso CH-PPS, planned for 2017. Participation was voluntary, and hospitals were paid CHF 200 (EUR 176) as a minimum fee and CHF 5 (EUR 4.40) per additional included patient exceeding the minimum fee, to improve participation. All patients hospitalised in acute care, regardless of medical specialty, were eligible if admitted to the ward before or at 8:00 and not discharged during the survey day. Patients in the emergency room, in psychiatry and in outpatient care were excluded.

### Data collection

The CH-PPS coordination centre organised seven interactive training workshops for hospital investigators and data collectors in three languages (German, French, and Italian) in March and April 2017. The courses followed a structured methodology, encouraging a participative, problem-solving approach by discussing clinical cases and using the database interactively. Patient data were collected on individual case report forms with demographic information and data on medical devices, antimicrobial use, HAI and microorganisms. Healthcare-associated infections were defined as outlined in the ECDC protocol version 5.3 [13]. Outcomes were stratified by hospital size (small: < 200 beds; medium-size: 200–650 beds; large: > 650 beds), hospital type (primary care, secondary care, tertiary care, specialised care), hospital ownership (public, private-not-for-profit, private-for-profit) and university-affiliation. The detailed methodology is described elsewhere [19].

Data collection started on 1 April 2017 and ended on 30 June 2017, with an advised collection window of no longer than 14 days. Data collection was done either by using the case report form followed by data entry into the electronic CH-PPS database or by direct data entry into the database. The format of the CH-PPS database featured plausibility algorithms to rule out missing data; it was provided by the Institute of Hygiene and Environmental Medicine, Charité University Medicine Berlin, Berlin, Germany [20]. Hospitals had the option to download their own data in different formats (HTML, CSV, pdf).

### Data validation

To assess the sensitivity and specificity of the PPS, a validation survey was performed in six hospitals, applying the ECDC protocol on data validation [13]: one university-affiliated hospital, two public medium-size hospitals, two public small hospitals and one private hospital. Three investigators from the CH-PPS coordination centre performed validation in 50 patients of each hospital, prioritising high-prevalence areas.

### Data analysis

Descriptive data are reported as medians with interquartile range (IQR) or means with 95% confidence intervals (CI), where appropriate. Statistical analysis of patient characteristics relative to hospital size was performed using the non-parametric Kruskal–Wallis test. A fatal McCabe score [21], age groups (0–17, 18–40, 41–60, 61–80, > 80 years), admission in an intensive care unit (ICU) on the survey day, exposure to a medical device (peripheral venous catheter, central venous catheter, urinary catheter, endotracheal tube) on the survey day, having undergone National Healthcare Safety Network (NHSN) surgery since hospital admission [22] and private-for-profit ownership were tested in a univariable logistic regression analysis as risk factors for HAI. Variables with a p value ≤ 0.2 were tested in a multivariable model. Observations were clustered on the hospital level and a two-sided p value of 0.05 was considered significant. Data analysis was performed using STATA version 13 (STATA Corporation). The HAI-incidence was estimated using the ECDC methodology based on the Rhame–Sudderth formula [12,23]. Median time from admission to survey data was used to estimate length of stay; median difference between length of stay and time from admission to first HAI was used to calculate the denominator [12]. Only the first HAI was taken into account.

### Benchmarking

A representative sample was obtained from the pooled data to allow benchmarking with the ECDC PPS data. Applying the ECDC methodology (design effect of 4.7, calculated from the data of the invited hospitals; precision of +/− 1%; HAI-prevalence of 5.9; the measured pooled prevalence; 95% CI), the randomised sample needed data from 56 hospitals, representing the distribution of hospital size in Switzerland. Six randomised sequences were generated, stratified by hospital size, using research randomiser (www.randomizer.org). The final sequence was randomly selected from the six previous sequences. To benchmark antimicrobial resistance (AMR), a composite index was calculated using the definitions of the second ECDC PPS [14]: number of first level AMR isolates, divided by the sum of the isolates for which results from antimicrobial susceptibility testing were reported. These first level markers were defined as *Staphylococcus aureus* resistant to meticillin (MRSA), *Enterococcus faecium* and *Enterococcus faecalis* resistant to vancomycin, Enterobacteriaceae resistant to third generation cephalosporins, and *Pseudomonas aeruginosa* and *Acinetobacter baumannii* resistant to carbapenems.

### Ethical statement

No institutional review board approval was deemed necessary, similar to the ECDC PPS, given the quality improvement character of the survey. Only anonymous patient and ward data were collected and analysed.

## Results

### Setting and study population

Ninety-six hospitals with 12,931 patients participated in the CH-PPS in 2017, 63 (66%) small hospitals with 3,516 (27.2%) patients, 26 (27%) medium-size hospitals with 4,380 (33.9%) patients and seven (7%) large hospitals with 5,035 (38.9%) patients. The survey covered 79% (68/86) of Swiss hospitals with 100 beds or more. All university-affiliated hospitals, but only two of the three free-standing children’s hospitals participated in the survey. One university hospital did not collect data on children. Sixty-eight participating hospitals (71%) had public, 14 (15%) private-not-for-profit and 14 (15%) private-for-profit ownership. Median duration of data collection was 2 days (IQR: 1–5), with three hospitals exceeding the recommended collection window of 14 days. The representative sample included 5,217 patients from two of seven large hospitals (7 invited hospitals), 10 of 26 medium-size hospitals (32 invited hospitals) and 44 of 63 small hospitals (148 invited hospitals). 

Table 1 summarises patient characteristics. Age, sex, rapidly fatal McCabe score, use of a vascular catheter and intubation differed significantly between hospitals of different size. Most patients were medical or geriatric (41.9%), surgical (36.1%) or obstetrical (7.9%). Median lengths of stay for large, medium-size and small hospitals, as calculated from annual admissions and patient days, were 8 days (IQR: 7–9), 7 days (IQR: 6–8) and 6 days (IQR: 5–7), respectively. Median time-to-survey for large, medium-size and small hospitals, as measured in the PPS, were 6 days (IQR: 2–15), 4 days (IQR: 2–9) and 3 days (IQR: 1–8), respectively.

**Table 1:** Patient characteristics – national point prevalence survey on healthcare-associated infections in acute care hospitals, Switzerland, 2017 (n = 12,931)

	All hospitals n = 12,931			Hospital size									p value
				< 200 beds n = 3,516			200–650 beds n = 4,380			> 650 beds n = 5,035			
	n	%	95% CI	n	%	95% CI	n	%	95% CI	n	%	95% CI	
Male sex	6,185	47.8	47.0–48.7	1,623	46.2	44.5–47.8	2,073	47.3	45.8–48.8	2,489	49.4	48.1–50.8	0.002
Age group													
0 years	509	3.9	3.6–4.3	147	4.2	3.5–4.8	161	3.7	3.1–4.2	201	4.0	3.5–4.5	0.501
1–17 years	481	3.7	3.4–4.0	214	6.1	5.3–6.9	82	1.9	1.5–2.3	185	3.7	3.2–4.2	< 0.001
18–40 years	1,647	12.7	12.2–13.3	475	13.5	12.4–14.6	512	11.7	10.7–12.6	660	13.1	12.2–14.0	0.033
41–60 years	2,284	17.7	17.0–18.3	568	16.2	14.9–17.4	737	16.8	15.7–17.9	979	19.4	18.4–20.5	< 0.001
61–80 years	4,942	38.2	37.4–39.1	1,250	35.6	34.0–37.1	1,795	41.0	39.5–42.4	1,897	37.7	36.2–39.0	< 0.001
> 80 years	3,068	23.7	23.0–24.5	862	24.5	23.1–25.9	1,093	25.0	23.7–26.2	1,113	22.1	21.0–23.5	0.002
McCabe score													
Not fatal	10,119	78.3	77.5–79.0	2,892	82.3	81.0–83.5	3,306	75.5	74.2–76.8	3,921	77.9	76.7–79.0	< 0.001
Ultimately fatal	1,730	13.4	12.8–14.0	456	13.0	11.9–14.1	611	13.9	12.9–15.0	663	13.2	12.2–14.1	0.903
Rapidly fatal	669	5.2	4.8–5.6	119	3.4	2.8–4.0	154	3.5	3.0–4.1	396	7.9	7.1–8.6	< 0.001
Unknown	413	3.2	2.9–3.5	49	1.4	1.0–1.8	309	7.1	6.3–7.8	55	1.1	0.8–1.4	0.008
Surgery and medical device use													
Surgery^a^	3,210	24.8	24.1–25.6	847	24.1	22.7–25.5	1,117	25.5	24.1–26.8	1,246	24.8	23.6–25.9	0.579
PVC	6,281	48.6	47.7–49.5	1,806	51.4	49.8–53.1	2,209	50.5	49.0–52.0	2,266	45.0	43.6–46.4	< 0.001
CVC	1,355	10.5	10.0–11.0	231	6.6	5.6–7.4	397	9.1	8.2–9.9	727	14.4	13.5–15.4	< 0.001
Urinary catheter	2,122	16.4	15.8–17.1	558	15.9	14.7–17.1	730	16.7	15.6–17.8	834	16.6	15.5–17.6	0.443
Intubation	212	1.6	1.4–1.9	42	1.2	0.8–1.6	58	1.3	1.0–1.7	112	2.2	1.8–2.6	< 0.001
Patient specialty													
Intensive care unit	531	4.1	3.8–4.4	124	3.5	2.9–4.1	154	3.5	3.0–4.1	253	5.0	4.4–5.6	< 0.001
Surgery	4,670	36.1	35.3–36.9	1,340	38.1	36.5–39.7	1,772	40.5	39.0–41.9	1,558	30.9	29.7–32.2	< 0.001
Medicine/geriatrics	5,415	41.9	41.0–42.7	1,368	38.9	37.3–40.5	1,944	44.4	42.9–45.9	2,103	41.8	40.4–43.1	< 0.001
Gynaecology	312	2.4	2.1–2.7	95	2.7	2.2–3.2	113	2.6	2.1–3.0	104	2.1	1.8–2.5	0.114
Obstetrics	1,021	7.9	7.4–8.4	329	9.4	8.4–10.3	316	7.2	6.4–8.0	376	7.5	6.7–8.2	< 0.001
Paediatrics	358	2.8	2.5–3.1	154	4.4	3.7–5.1	61	1.4	1.0–1.7	143	2.8	2.4–3.3	< 0.001
Other specialty	624	4.8	4.5–5.2	106	3.0	2.4–3.6	20	0.5	0.3–0.7	498	9.9	9.1–10.7	< 0.001

CI: confidence interval; CVC: central venous catheter; PVC: peripheral venous catheter.

^a^Surgery since admission; national safety healthcare network surgery only [22].

### Prevalence of healthcare-associated infections

A total of 765 patients had 835 HAI. The pooled HAI-prevalence was 5.9% (765/12,931; 95% CI: 5.5–6.3). The pooled prevalence of HAI attributable to the hospital and occurring during the current stay was 5.2% (672/12,931; 95% CI: 4.8–5.6%) and 4.2% (547/12,931; 95% CI: 3.9–4.6), respectively. Male sex (448/6,185; 7.2%; 95% CI: 6.6–7.9), a rapidly fatal McCabe score (71/669; 10.6%; 95% CI: 8.3–13.0) and age > 40 years (690/10,294; 6.7%; 95% CI: 6.2–7.2) were groups with statistically significantly higher HAI prevalences. The estimated incidence was 4.5% (95% CI: 4.0–5.0). The representative HAI prevalence was 5.4% (282/5,217; 95% CI: 4.8–6.0). The representative prevalence of HAI attributable to the hospital and occurring during current stay was 4.6% (242/5,217; 95% CI: 4.1–5.2) and 3.6% (185/5,217; 95% CI: 3.0–4.0), respectively. The adjusted prevalence taking into account the results from the validation study was 7.2% (95% CI: 4.5–10.5).

**Figure 1:**
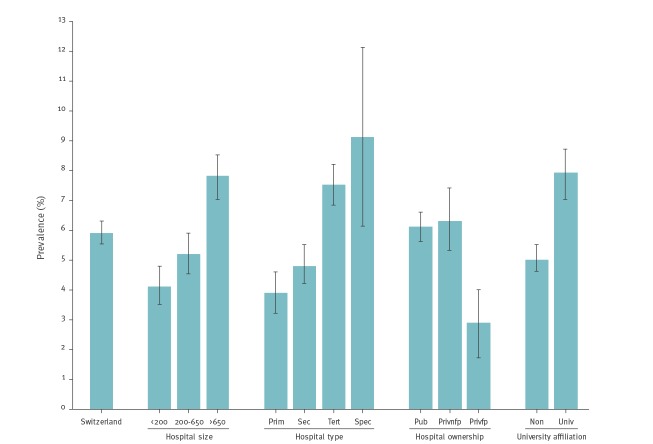
Prevalence of healthcare-associated infections, stratified by hospital risk factors, Switzerland, 2017 (n = 765)

Figure 1 summarises pooled HAI prevalences stratified by hospital characteristics. Large (392/5,035; 7.8%; 95% CI: 7.0–8.5), tertiary care (418/5,549; 7.5%; 95% CI: 6.8–8.2) and specialised care (33/363; 9.1%; 95% CI: 6.1–12.1) hospitals, hospitals with a public mandate (741/12,091; 6.1%; 95% CI: 5.7–6.6) and university-affiliated hospitals (321/4,087; 7.9%; 95% CI: 7.0–8.7) had significantly higher HAI prevalences.

Hospital size: < 200 beds (small), 200–650 beds (medium-size), > 650 beds (large); hospital type: prim (primary care), sec (secondary care), tert (tertiary care), spec (specialised hospital); hospital ownership: pub (public), privnfp (public-not-for-profit), privfp (private-for-profit); university affiliation: non (not affiliated), univ (affiliated).

Bars and whiskers indicate the HAI prevalences with 95% confidence intervals.

**Figure 2:**
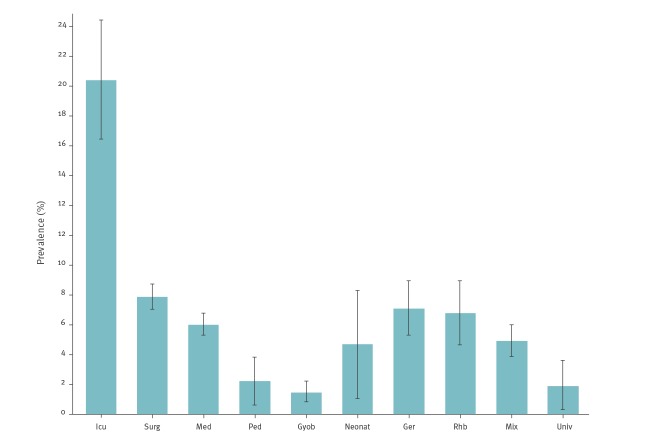
Prevalence of healthcare-associated infections, stratified by ward type, Switzerland, 2017 (n = 765)

Figure 2 summarises the pooled HAI prevalences stratified by ward specialty. The highest HAI prevalence was observed in intensive care (97/471; 20.6; 95% CI: 16.9–24.3). Prevalences in surgery (264/3,669; 7.2%; 95% CI: 6.4–8.0), medicine (210/3,823; 5.5%; 95% CI: 4.8–6.2), geriatrics (52/801; 6.5%; 95% CI: 4.8–8.2) and rehabilitation (35/561; 6.2%; 95% CI: 4.2–8.2) were in a similar range. Prevalences in gynaecology/obstetrics (16/1,212; 1.3%; 95% CI: 0.7–2.0) and paediatrics (7/349; 2.0%; 95% CI: 0.5–3.5) were low.

Ger: geriatrics; gyob: gynaecology and obstetrics; icu: intensive care unit; med: medicine; mix: mixed ward (< 80% of one specialty); neonat: neonatology; other: other ward type; ped: paediatrics; rhb: rehabilitation; surg: surgery.

Bars and whiskers indicate the HAI prevalences with 95% confidence intervals.

### Distribution of healthcare-associated infections

**Figure 3:**
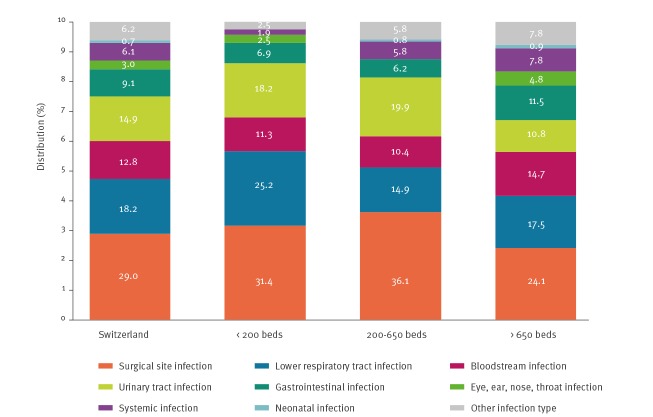
Distribution of healthcare-associated infections, stratified by hospital size, Switzerland, 2017 (n =835)

Figure 3 summarises the distribution of the main HAI types stratified by hospital size. The highest proportion were SSI (242/835; 29.0%; 95% CI: 25.9–32.1), followed by lower respiratory tract infections (LRTI) (152/835; 18.2%; 95% CI: 15.6–20.8), UTI (124/835; 14.9%, 95%CI: 12.4–17.3%), BSI (107/835; 12.8%; 95% CI: 10.5–15.1) and gastrointestinal infections (GI) (76/835; 9.1%; 95% CI: 7.1–11.1). Of the 76 episodes of GI, 36 (47.4%) were due to *Clostridium difficile*, yielding a prevalence of *C. difficile* infection of 0.3% (36/12,931; 95% CI: 0.2–0.4) and accounting for 4.3% (36/835) of all HAI.

Hospital size: < 200 beds (small), 200–650 beds (medium size), > 650 beds (large).

**Figure 4:**
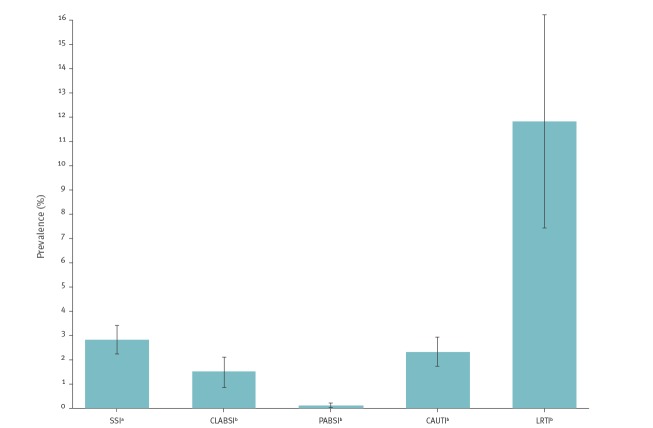
Prevalence of device-associated and surgical site infections, Switzerland, 2017 (n = 190)

CAUTI: catheter-associated urinary tract infection; CLABSI: central line-associated bloodstream infection; LRTI: lower respiratory tract infection; PABSI: peripheral catheter-associated bloodstream infection; SSI: surgical site infection.

^a^ Denominator: number of national healthcare safety network surgeries during current hospital stay.

^b^ Number of infections as per patients having a relevant medical device in place at survey.

Figure 4 summarises the prevalences of device-associated HAI and SSI. The prevalence of LRTI among patients on ventilation on the survey day was highest (25/212; 11.8%; 95% CI: 7.4–16.2), followed by UTI among patients exposed to a urinary catheter on the survey day (49/2,122; 2.3%; 95% CI: 1.7–2.9) and catheter-associated infections among those exposed to a central venous catheter on the survey day (20/1,355; 1.5%; 95% CI: 0.8–2.1). The prevalence of catheter-associated infections among patients with a peripheral venous catheter in place on the survey day was low (6/6,281; 0.1%; 95% CI: 0.0–0.2). The prevalence of SSI after NHSN surgery was 2.8% (90/3,210; 95% CI: 2.2–3.4). The prevalence of any type of HAI among patients undergoing NHSN surgery was 8.8% (281/3,210; 95% CI: 7.8–9.7).

Bars and whiskers indicate the HAI prevalences with 95% confidence intervals.

### Isolated microorganisms from healthcare-associated infections

A total of 69.0% of the infections (576/835; 95% CI: 65.7–72.1) were microbiologically investigated, of which 93.1% (536/576; 95% CI: 90.7–95.0) were positive for 746 microorganisms. Table 2 summarises the distribution of the major groups of microorganisms. A total of 59 (23.0%; 95% CI: 17.8–28.1) of the 257 enterobacteria had reduced susceptibility to third-generation cephalosporins and five (1.9%; 95% CI: 0.2–3.6e) had reduced susceptibility to carbapenems; 11 (11.0%; 95% CI: 4.8–17.2) of the 100 *S. aureus* were resistant to meticillin; two (2.2%; 95% CI: 0.0–5.3) of the 90 enterococci were resistant to vancomycin; and five (11.4%; 95% CI: 1.6–21.1) of the 44 *P. aeruginosa* were resistant to carbapenems. The composite index for Switzerland was 15.6% (77/494; 95% CI: 12.4–18.8).

**Table 2:** Major groups of microorganisms, stratified by type of healthcare-associated infection, Switzerland, 2017 (n = 746)

	SSI		UTI		BSI		LRTI		GI		Other		Total	
	n	%	n	%	n	%	n	%	n	%	n	%	n	%
Gram-positive cocci	149	49.7	22	18.5	68	61.3	19	24.1	13	16.3	29	50.9	300	40.2
Gram-positive bacilli,	4	1.3	0	0.0	2	1.8	0	0.0	1	1.3	1	1.8	8	1.1
*Enterobacteria*	86	28.7	83	69.8	30	27.0	34	43.0	15	18.8	9	15.8	257	34.5
Gram-negative bacilli	20	6.7	12	10.1	3	2.7	17	21.5	2	2.5	6	10.5	60	8.0
Gram-negative cocci	1	0.3	0	0.0	1	0.9	2	2.5	0	0.0	0	0.0	4	0.5
Anaerobic bacilli	17	5.7	0	0.0	1	0.9	1	1.3	36	45.0	2	3.5	57	7.6
Other bacteria	3	1.0	0	0.0	0	0.0	0	0.0	0	0.0	2	3.5	5	0.7
Fungi	20	6.7	2	1.7	6	5.4	2	2.5	11	13.8	4	7.0	45	6.0
Viruses	0	0.0	0	0.0	0	0.0	4	5.1	2	2.5	4	7.0	10	1.3
**Total**	**300**	**100.0**	**119**	**100.0**	**111**	**100.0**	**79**	**100.0**	**80**	**100.0**	**57**	**100.0**	**746**	**100.0**

BSI: bloodstream infection; GI: gastrointestinal infection; LRTI: lower respiratory tract infection; Other: other types of healthcare-associated infections; SSI: surgical site infection; UTI: urinary tract infection.

### Risk factors for healthcare-associated infections

Table 3 summarises uni- and multivariable testing of risk factors for HAI. The HAI prevalence was significantly higher in large hospitals, tertiary care hospitals, intensive care, patients with a fatal McCabe score, male patients and patients at older age. The lower HAI prevalence in private-for-profit hospitals (2.9%; 95% CI: 1.7–4.0) was not statistically significant compared with public hospitals and private-not-for-profit hospitals in the multivariable analysis.

**Table 3:** Overall risk factors for healthcare-associated infections, Switzerland, 2017 (n = 765)

Variable	Univariable analysis			Multivariable analysis		
	OR	95% CI	p value	OR	95% CI	p value
Large hospitals^a^	1.70	1.27–2.28	< 0.001	1.33	1.07–1.66	0.011
Tertiary care	1.65	1.24–2.20	0.001	1.23	1.00–1.52	0.045
Private-for-profit^b^	0.45	0.29–0.70	< 0.001	0.63	0.39–1.02	0.059
Intensive care unit	4.58	3.53–5.93	< 0.001	4.17	3.13–5.56	< 0.001
Fatal McCabe score^c^	2.01	1.66–2.43	< 0.001	1.68	1.40–2.03	< 0.001
Male sex	1.58	1.38–1.81	< 0.001	1.45	1.29–1.64	< 0.001
Age group^d^	1.20	1.14–1.26	< 0.001	1.18	1.11–1.25	< 0.001

CI: confidence interval; OR: odds ratio.

^a^ Large hospitals: hospitals > 650 beds.

^b^ Private-for-profit: private-for-profit hospitals compared with other hospital ownerships (public, private-not-for-profit).

^c^ Fatal McCabe score: ultimately and rapidly fatal McCabe scores combined.

^d^ Age groups: 0 years, 1–17 years, 18–40 years, 41–60 years, 61–80 years, > 80 years.

### Benchmarking to the second ECDC point prevalence survey

**Figure 5:**
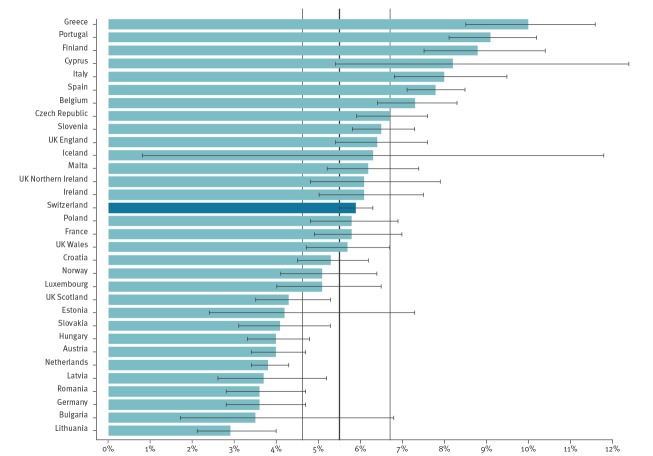
Prevalence of patients with healthcare-associated infections in the Swiss and the ECDC point prevalence surveys combined

CI: confidence interval; ECDC: European Centre for Disease Prevention and Control; HAI: healthcare-associated infections.

Given that Switzerland used the ECDC protocol and conducted the CH-PPS at the same time as the second ECDC PPS, data could be benchmarked to the EU and EEA countries. Figure 5 shows the position of Switzerland compared with the pooled PPS results of the second ECDC PPS [14]. 

Bars and whiskers: HAI prevalences and 95% CI by country. Vertical lines: mean prevalence and 95% CI across the countries participating in the 2016 and 2017 ECDC point prevalence survey. Source: [14].

## Discussion

This was the first point prevalence, and by the number of participating hospitals and patients, the largest national HAI prevalence survey in Switzerland. Participation of almost 80% of the acute care hospitals with 100 beds or more renders this survey highly representative for Switzerland. The pooled HAI prevalence (5.9%) was similar to the average European HAI prevalence in the first ECDC PPS of 2011 and 2012 (6.0%) [12] and higher than that in the second ECDC PPS of 2016 and 2017 (5.5%; 95% CI: 4.6–6.7) [14]. Similar ratios were measured in France (5.8%; 95% CI: 4.9–7.0), Ireland (6.1%; 95% CI: 5.0–7.6), Poland (5.8%; 95% CI: 4.8–6.9), England (6.4%; 95% CI: 5.4–7.6), Northern Ireland (6.1%; 95% CI: 4.8–7.9) and Wales (5.7%; 95% CI: 4.7–6.7) in 2016 and 2017. Ratios in Austria (4.0%; 95% CI: 3.4–4.7) and Germany (3.6%; 95% CI: 2.8–4.7) were statistically significantly lower; the ratio in Italy (8.0%; 95% CI: 6.8–9.5) was statistically significantly higher. 

The past HAI prevalence surveys in Switzerland used the period methodology and thus, comparison with historical data is difficult. However, it has been estimated that, compared with the point methodology, the period methodology inflates the HAI prevalence by 32% [8]. Thus, the reported period prevalence of 7.2% in the most recent national survey from 2004 would translate to a point prevalence around 5.5%. We may therefore hypothesise that the HAI prevalence did not change in Swiss acute care hospitals in the past years. The stable course of the yearly period prevalence surveys at the University of Geneva Hospitals between 2006 and 2013 further supports this hypothesis [8]. Switzerland established a national strategy aiming at HAI prevention only in 2016 [18]. At the same time, Swissnoso initiated the first HAI strategy aiming at SSI prevention [24]. This ongoing programme is the first national HAI initiative in Switzerland since the hand hygiene campaign in 2006 [25], which was part of the World Health Organization “Clean Care is Safer Care” campaign launched in 2005 [26,27].

With a proportion of 29%, SSI was the most common HAI. This is substantially higher compared with the ECDC PPS, where the proportion of SSI among HAI was 19.6% in 2011 and 2012 and 18.4% in 2016 and 2017 [12,14]. This difference can be explained in part by the fact that the proportion of both surgical patients and patients undergoing NHSN surgery was higher in the CH-PPS than in Europe in 2011 and 2012 (36.1% vs 24.8 and 30.6% vs 20.2%, respectively) [12]. The HAI prevalence among patients undergoing NHSN surgery was even lower in the Swiss PPS (8.8%) than in Europe in 2011 and 2012 (10.1%) [12]. The lower HAI prevalence in private-for-profit hospitals was due to more favourable risk factors such as lower age, more favourable McCabe scores and shorter length of stay before survey. Gram-positive cocci were the most common pathogens in the CH-PPS (40.2%), followed by enterobacteria (34.5%), with *E. coli* and *Klebsiella* spp. together representing 22.5% of all isolates. This proportion is similar to the findings of the first ECDC PPS (36.2% for all enterobacteria; 24.6% for *E. coli* and *Klebsiella* spp. together), the second ECDC PPS (26.5% for *E. coli* and *Klebsiella* spp. together), the PPS from the US CDC (19.6% for *E. coli* and *Klebsiella* spp. together) and the last Swiss period prevalence survey in 2004 (23.0% for *E. coli* and *Klebsiella* spp. together) [12,14,16,28]. The AMR composite index of 15.5% in Switzerland was lower compared with the second ECDC PPS with 31.6% [14], ranking in the lower range similar to Austria (12.4%), the Netherlands (14.5%), and Luxemburg (14.9%). The proportion of carbapenem-resistant enterobacteria was much lower than in the second ECDC PPS (6.2%) [14]. While *C. difficile* contributed 12.1% in the most recent US CDC PPS, its role in Switzerland was much smaller (4.7%) [28], similar to the second ECDC PPS in 2016 and 2017 (4.9%) [14]. In the absence of a national surveillance system for the incidence of *C. difficile* infection in Switzerland, this finding must be interpreted with caution.

Our survey has limitations. Firstly, the prevalence is a point estimate, and deduction of the burden of HAI is not suitable, particularly because of the length bias caused by over-representation of patients with increased length of stay [29]. However, even if a point estimate is limited for an individual hospital, on a national basis, the results can be considered valid and the participation of 80% of Swiss acute care hospitals with 100 or more beds makes this study representative of the burden of HAI in Switzerland. Secondly, variation in the application of HAI definitions was likely despite multiple centralised workshops for hospital data collectors. This is evidenced by the results of the validation study, which resulted in an unexpected high adjusted HAI prevalence. Thirdly, significant differences in healthcare practices and lengths of stay in Europe limit comparison of HAI between Switzerland and the EU/EEA countries. Finally, compared with Switzerland as a whole, medium-size and large hospitals were over-represented in our database, and thus, the measured HAI prevalence may have been overestimated. This was addressed by analysing a randomised subsample, taking into account the size distribution of acute care hospitals in Switzerland.

From an organisational perspective, the survey was successful in achieving the desired objectives: engaging the majority of Swiss acute care hospitals in a national project, achieving good data representativeness, updating national data on HAI given that the last period-prevalence in Switzerland was performed in 2004 and, for the first time, generating data that are comparable with other countries in Europe.

## Conclusions

This first national point-prevalence survey in Switzerland identified an HAI prevalence at European average. Compared with previous national surveys, which used a different methodology, it still can be estimated that the HAI prevalence in Switzerland was stable in the past decade. Given the unchanged situation in Switzerland with a higher prevalence compared with its geographical neighbours, efforts in HAI prevention should be established.
